# Riboflavin Transporter Deficiency as a Cause of Progressive Encephalopathy

**DOI:** 10.3390/metabo15110688

**Published:** 2025-10-24

**Authors:** Justyna Paprocka, Julia Karpierz, Michał Hutny, Jagoda Hofman-Hutna, Artur Dobosz

**Affiliations:** 1 Department of Pediatric Neurology, Faculty of Medical Sciences in Katowice, Medical University of Silesia, 40-752 Katowice, Poland; 2 Students’ Scientific Society, Department of Pediatric Neurology, Faculty of Medical Sciences in Katowice, Medical University of Silesia, 40-752 Katowice, Poland; 3 Department of Medical Genetics, Faculty of Medicine, Jagiellonian University Medical College, 31-008 Krakow, Poland; artur.dobosz@uj.edu.pl

**Keywords:** RTD2, WES, motor delay, ataxia

## Abstract

**Background/Objective:** Riboflavin transporter deficiency (RTD) is a rare neurodegenerative disease, with under 500 cases genetically confirmed since the early 2000s. Thus far, three separate subtypes of RTD2 are described—type 1, 2 and 3—but, previously, RTD was classified as two separate genetic defects: Brown–Vialetto–Van Laere syndrome and Fazio–Londe syndrome, caused by mutations in the *SLC52A2* and *SLC52A3* genes, respectively. The most prominent symptoms found in patients include encephalopathy, expressed as peripheral and cranial nerve neuropathy, which in turn lead to a series of complications: decreased muscle strength, hypotonia, visual impairment, sensorineural hearing loss, bulbar palsy, sensory ataxia and respiratory insufficiency secondary to diaphragmatic paresis. At the cellular level, riboflavin is modified into active flavin cofactors: FMN, mediating riboflavin phosphorylation through riboflavin kinase, and FAD, involved in FMN adenylation through the flavin dinucleotide 1 synthesis. FMN and FAD are two of approximately 100 proteins collectively described as the ‘flavoproteome’. Most of them are mitochondrial oxidoreductases, catalyzing the electron transport in many metabolic reactions, as well as regulating important cell processes, such as the production of reactive oxygen species, protein conformation and damage repair. FMN and FAD are also responsible for the conversion of B6 and B9 vitamins into their active forms, which allows for healthy cell growth and immune function. **Methods:** In this article, the authors describe two children, a 6-year-old girl and her 5-year-old sister, both presenting with RTD2 caused by mutations in the *SLC52A2* gene (c.916G>C (p.Gly306Arg); c.477C>G (p.Cys159Trp)), in whom the disease progression was successfully inhibited by vitamin B_2_ supplementation in varying doses. **Results:** Their clinical image consists of psychomotor developmental delay, ataxia, horizontal nystagmus, hearing loss and a lack of visual fixation. **Conclusions:** The phenotype and clinical signs presented by the described sisters are further discussed in relation to the previously published reports of RTD2 cases.

## 1. Introduction

Vitamin B_2_, also called riboflavin, is one of the crucial microelements in the human diet. Its recommended intake depends on various factors (such as age, gender, metabolic activity) and ranges from 0.3 mg for infants to 1.6 mg for breastfeeding women [1,2], with the consensus being 1.3 mg/day for men and 1.1 mg/day for women. For children, the recommended daily intake is extrapolated down from adult dosages, taking into consideration body weight and age, and usually ranges from 0.5 to 0.6 mg/day in children aged 1–9 and between 0.9 and 1.3 mg/day in adolescents (ages 10–18) [3,4]. The direct function of this vitamin is deceivingly narrow—it serves as a substrate for the synthesis of two coenzymes: flavin mononucleotide (FMN) and flavin adenine dinucleotide (FAD) [5,6]. These in turn are cofactors for a wide range of enzymes, collectively described as flavoproteins [2,6]. Therefore, vitamin B_2_ indirectly influences numerous processes in human organisms, including the mitochondrial electron transport chain, cholesterol [7] and essential fatty acid biosynthesis [2,5,6,8,9,10].

As riboflavin cannot be synthesized by human cells, providing it in the diet and proper absorption are necessary for maintaining its optimal levels. In developed countries, vitamin B_2_ deficits are relatively rare, though some specific groups might be susceptible due to nutritional or clinical factors, especially older adults, alcoholics, vegans and women using oral contraceptives [1]. For this reason, several foods that are naturally high in riboflavin were identified, and their introduction to the diet, or increasing their consumption, is recommended for those groups by physicians. In the Western world, dairy products and milk are the biggest contributors to daily vitamin B_2_ intake, as well as various meats and oily fish. Another source, especially important for vegetarians and vegans, is dark green vegetables such as spinach, as well as broccoli and asparagus [11]. Although grain products do naturally contain a medium-to-low concentration of riboflavin, most of it is lost after processing. For this reason, in many countries (such as the United States of America, Canada, Guatemala and others), cereal grains, wheat flour, corn flour and other grain-derived products are additionally fortified with riboflavin, whereas some countries, like India, have a voluntary vitamin B_2_ fortification program [11,12,13].

Nevertheless, riboflavin deficiency may also result from genetic defects, among which a distinctive group is riboflavin transporter deficiencies (RTDs). They were previously known as Brown–Vialetto–Van Laere syndrome 2 (21BVVLS2; #614707), in which mutations in the *SLC52A2* (8q24.3; #607882) or *SLC52A3* (20p13; #613350) genes were responsible for the disease occurrence, and as Fazio–Londe disease (FLD; #211500), caused solely by mutations in the *SLC52A3* gene [14]. Thus far, RTDs are classified with numbers 1, 2 and 3, with the affected genes being *SLC52A1*, *SLC52A2* and *SLC52A3*, respectively [15,16].

The estimated prevalence of RTD2 is less than 1 per 1,000,000; however, due to its rarity and complex symptomatology, the exact number is difficult to estimate due to the probable underdiagnosis of the disease [15].

These neurometabolic diseases may present a wide range of symptoms, resulting from encephalopathic cranial, motor (80% of patients) and sensory (34% of patients) neuropathies. Even though RTD may present in different stages of life [14], affected individuals are most likely to experience symptoms from early childhood. The pathogenesis of these disorders is not yet determined, but is thought to concern the influence of riboflavin transporters on mitochondria. The lacking activity of these proteins leads to deficits in bioenergetics, impaired peroxisomal and mitochondrial biogenesis, oxidative stress and impaired synaptic transmission [17]. The neuropathies mentioned above may lead to mild or more severe symptoms, ranging from decreased muscle strength, hypotonia, facial nerve palsy, visual impairment and gait ataxia to sensorineural deafness, bulbar palsy, severe disability and respiratory failure [14,18]. A review of the available data indicated that life expectancy in patients without adequate treatment is under 12 years. The mean time between the emergence of first symptoms and death does not exceed 5 years [19,20,21]. Currently, few studies report cases of patients who lived until old age [20,21].

Though RTDs are classified as rare, and the reports in the available literature are relatively scarce, the currently recommended management strategy is, in most cases, a supplementation of vitamin B_2_. This treatment presents a very promising efficiency and is therefore regarded as life-saving [18,22,23,24]. In accordance with CARE guidelines [25,26], this article gives important insight into the treatment experiences of two girls with RTD2, a 6-year-old (Patient 1) and her 5-year-old sister (Patient 2), which led to a moderate improvement of the patients’ conditions. Though the progression of the disease was inhibited, some of the pathological features, such as generalized hypotonia and sensory impairment, remained persistent in both girls, though their development was limited in different extents. The discussion in relation to previous case reports found in the literature concerning the successfulness and safety of riboflavin supplementation in RTD2 patients is presented in the later part of the article.

## 2. Case Presentation

### 2.1. Patient Information

Patient 1 is a 6-year-old girl born to non-consanguineous parents, whose prenatal, perinatal and early childhood medical history was insignificant. At the age of three, she started to experience some symptoms of impaired mobility and coordination, first presenting as minor stumbles that later evolved into falling down and ataxia of the upper extremities and trunk, as well as right-sided knee pain and increased weariness. Her visual acuity was limited due to progressive myopia, as in case of her brother and grandmother, though, at the start of the symptoms the patient has not yet been consulted ophthalmologically. At this time, she was relatively small for her age (91 cm; 3–10 centile) and weighed ca. 12 kg (3–10 centile), which amounted to a body mass index (BMI) of 14.67. Her head circumference (35 cm) at birth was within a normal range.

Patient 2 is the younger, 5-year-old sister of Patient 1 from the same parents. She was born at 39 gestational weeks after the pregnancy was complicated by group B Streptococcus (GBS) infection, polycythemia vera and a two-vessel umbilical cord. Nevertheless, her birth weight, length and head circumference were within the physiological range, and she scored 10 points on the Apgar scale. In infancy, the patient exhibited sensory disorders within the stomatognathic system, such as the biting and clenching of the teeth, as well as a high hypersensitivity to gentle touch. In addition, her parents observed a strong C-straightening and frequent sleeping with widespread legs. Her further development was delayed in terms of motor functions. Though eventually she learned to walk at the age of 20 months, her gait was unstable, leading to frequent falls.

### 2.2. Clinical Findings

The neurological examination of Patient 1 conducted during the first admission to the pediatric neurology ward confirmed the previously observed gross and fine motor deficits, further detecting hypotonia, absent reflexes in the lower extremities and hyporeflexia in the upper extremities. The patient’s speech was also affected, though the introduced therapy returned positive results, as it did with the other symptoms (to a greater or lesser extent).

A week later, the patient was again admitted to the same hospital with the onset of new symptoms: dysmetria, horizontal nystagmus, hyperextension in the knee joints and hyporeflexia of the superficial reflexes. According to her parents, the patient’s visual acuity and hearing worsened significantly. A right-sided functional scoliosis was observed. The patient’s condition declined further with the course of the disease, as her initial symptoms were soon accompanied by episodic urine incontinence and dysphagia. The ataxic features were more prominent on the left side.

Despite myopia, which significantly limited the patient’s visual acuity, the consulting ophthalmologist observed no significant structural pathologies in both eyes. Defects of the cardiovascular system, apart from the enlarged left ventricle, were ruled out after a series of functional and imaging examinations.

The initial presumptive diagnosis was myeloencephalitis of unclear etiology, though Guillain–Barré syndrome was also taken into consideration. In the meantime, the patient was also tested for SARS-CoV-2 due to the reported symptoms of infection, though the results were negative. She was discharged from infectious diseases and referred back to the neurology ward. The genetic assessment for cerebellar–spinal ataxia was conducted, targeting primarily CLN2-associated disorders, despite the lack of apparent myoclonia, as well as Niemann–Pick disease type C, though neither hepatosplenomegaly nor paresis of vertical eye movements was observed.

Patient 2 presented a similar, though less extensive phenotype of neurological symptoms. During an examination, the axial muscle tone was lowered, with alternating tonus peripherally and absent tendon reflexes in the lower extremities. Ataxia was observed within the trunk and extremities. She tested positive in Romberg’s test, falling down backward. From the age of 9 months, snoring was noticed, which might be suggestive of a dysfunctional pharyngeal musculature. Consistent with the symptomatology of Patient 1, hypermobility and hyperlaxity in the joints of lower extremities, as well as pelvic anteversion, were observed.

### 2.3. Diagnostic Assessment

Taking into consideration the wide spectrum of symptomatology presented by the described patients, a precise and multidirectional assessment was an obvious necessity, consisting of both functional and imaging examinations, as well as eventual genetic testing using whole exome sequencing.

The two primarily used imaging methods were magnetic resonance imaging (MRI) of the brain, spine and abdomen and abdominal and pulmonary ultrasonography (USG). The images of abdominal structures raised no suspicion, and neither did the USG image of the lungs. Similarly, abdominal USG and echoencephalography in Patient 2’s case detected no pathologies. Though initially post-inflammatory alterations were observed in the lumbosacral part of the meningeal sac, presumably affecting the spinal nerve roots, no such observation was made in the imaging conducted after the following month. Apart from a 5 mm pineal gland cyst and a minor enlargement of the right lateral ventricle, no pathologies in terms of brain structure morphology were discovered in the T1, T2, DWI and FLAIR MRI sequences.

In order to properly assess the pathomechanism of muscular hypotonia, as well as the ataxic features observed in the patient, electroneurography was conducted. Its results showed a physiological response of motor fibers, whereas the amplitude of the response to sensory stimuli was lowered in bilateral sural nerves and the right medial nerve. Those findings were interpreted as corresponding with the characteristics of diffuse axonal damage in the sensory fibers of peripheral nerves.

Another functional assessment applied in the described case was electroencephalography, conducted during sleep and in wakefulness. Though the latter detected no abnormalities, the sleep electroencephalography trace showed a generalized photosensitive paroxysmal activity.

In order to properly assess the issue of developing hearing impairment, auditory evoked potentials were examined, but the results proved to be bilaterally physiological. The patient was eventually diagnosed with sensorineural hearing loss. An attempt to assess visually evoked potentials failed due to the patient’s anxiety and lack of cooperation.

Laboratory examinations were also useful in the diagnostic process, but rather in terms of excluding the potential pathomechanisms than explaining the symptoms themselves. The patient was examined hormonally for thyroid function, as well as for the status of vital microelements such as iron, copper and vitamins: B_12_, B_9_, D, E, folic acid and biotinidase. These methods were also useful in screening for metabolic defects, such as lysosomal storage diseases, though similarly to other laboratory results these also failed to produce any explanation for the patient’s condition.

Despite all examinations, the decisive method was genetic assessment. Initial tests for spinocerebellar ataxia using next-generation sequencing (NGS) returned no significant results. By conducting whole exome sequencing in Patient 1, a pathogenic mutation in the *SLC52A2* gene (c.916G>C (p.Gly306Arg); c.477C>G (p.Cys159Trp)) was discovered, allowing for the final diagnosis—RTD2. The first mutation is a pathogenic missense variant (Franklin ACMG Classification, Varsome: Pathogenic, MutationTaster: disease causing prob:0.99999), and it is a frequent finding among RTD2 patients and, in the current case, was inherited from the father, whereas the latter mutation (c.477C>G) is classified as a VUS (variant of uncertain significance, Varsome: Likely Pathogenic, MutationTaster: disease causing prob: 0.9999975) and was inherited from the mother. The segregation of the variant c.477C>G in the family gives us a strong suspicion that this variant is probably pathogenic and, in correlation with the variant c.916G>C, is the cause of the clinical symptoms.

Additionally, during the whole exome sequencing test in Patient 1, the mitochondrial genome was also assessed, and a variant of uncertain significance was found (Varsome: Likely Benign), m.9098T>C, p.Ile191Thr in the *MT-ATP6* gene in heteroplasmy (in 145 reads of 709 reads).

In Patient 2, the same variants in the *SLC52A2* gene (c.916G>C (p.Gly306Arg); c.477C>G (p.Cys159Trp)) were confirmed using the Sanger sequencing method (Figure S1). Also, variant m.9098T>C, p.Ile191Thr in the *MT-ATP6* gene was confirmed, but we could not assess the state of heteroplasmy.

The clinical symptoms in both patients could also suggest mitochondrial disorder, but the variant in the *MT-ATP6* gene is probably benign and only family members with two variants in the *SLC52A2* gene have neurological symptoms.

### 2.4. Therapeutic Intervention

#### 2.4.1. Nutrition and Feeding

After a successful genetic assessment and a final diagnosis of RTD2, Patient 1 received an appropriate treatment, riboflavin supplementation, which was also introduced as a preventive measure in Patient 2 An initial dose of vitamin B_2_ was 50 mg/kg, divided into four daily doses taken orally together with meals, which is necessary for the better absorption of riboflavin from the intestines. By the end of the first month of therapy, the dose was increased to 75 mg/kg in Patient 1, which led to significantly improved energy levels. Eventually, a year later, the doses were again increased to 100 mg/kg due to SARS-CoV-2 infection, with shortened intervals between the doses (from every 6 h to every 4 h).

Apart from the riboflavin supplementation, the patients receive the so-called ‘mitochondrial cocktail’, which is a part of the treatment used in many RTD patients around the world, consisting of coenzyme Q10 (ubichinon or ubichinol), B-complex and vitamins C and E, as well as L-carnitine, lipoic acid and N-acetylcysteine, the latter of which is currently not used [27].

#### 2.4.2. Hearing Impairment

Due to the severe bilateral sensorineural hearing loss found in Patient 1, a decision was made to implant cochlear implants (Medel) into the right and left ear. In neither of these operations did the patient experience any adverse events, both intra- and perioperatively. An improvement was observed in the reduction in the hearing threshold from >90 dB (binaural deep neurosensory hearing loss) before the implantation to the threshold of 60–45 dB (moderate hearing loss) after the implantation. Despite the surgery and rehabilitation, the patient does not differentiate between speech and sound, which in turn impairs her ability to develop her own speech; as a result, the parents decided to use augmentative and alternative communication.

### 2.5. Follow-Up and Outcomes

After the start of riboflavin supplementation, the condition of Patient 1 improved significantly over a short period of time in many aspects. In the first four months of treatment, an increased muscular strength in the neck and arm musculature was observed, as well as improved energy levels. Nevertheless, some pathological features remained persistent, especially in terms of general hypotonia, peripherally more prominent in the upper extremities. Similarly, the hypermobility of joints is still present, especially in elbow and wrist joints. However, that is not a big concern, as Patient 1 prefers the pathological wrist extension to the physiological wrist flexion. Due to the muscular atrophy of wrist extensor muscles, the antagonistic flexor muscles became shortened, ultimately leading to finger contracture.

The progressive neurogenic scoliosis (Cobb angle > 70°) in Patient 1 requires an operative treatment—the proposed solution is a vertebral operation with magnetic rods. It is an important medical issue, as scoliosis has an impact on the development of gross and fine motor skills in patients. Furthermore, the girl is able to walk only with assistance. The difficulties in psychomotor development stem in a high measure from the communicative barrier, which in turn is influenced by the hearing impairment of the patient. The patient was and still is currently attending physiotherapist visits and rehabilitation a couple of times a week.

The condition of Patient 2 also improved significantly due to the riboflavin therapy, though some of its features, such as ataxic gait and scoliosis (Cobb angle—30°) remained persistent. The speech development in this patient is physiological, though interdental lisping was observed. It is not, however, an unsettling finding, as this type of speech impairment is frequent among children her age.

The treatment, however, led also to some adverse events—shortly after the initiation of the therapy, the dysphagia of Patient 1 worsened, leading to rapid weight loss, which was corrected to the initial weight over the span of six months. The patient’s dysphonia also evolved into an aphonia lasting two months. The supplementation also triggered excessive salivation, dyspepsia and body temperature dysregulation: night sweats and alarming blue spells.

## 3. Discussion

The disorders associated with genetically determined RTD, which include RTD2, present with a variety of neurological symptoms [6,10,28]. These are naturally caused by the general pathophysiological mechanisms consisting of oxidative stress, bioenergetic deficits and impaired mitochondrial biosynthesis, which consequently lead to axonal neuropathy. This process is not selective for any specific region of the nervous system, but rather concerns every neuronal pathway [29]. That mechanism is responsible for a rich symptomatologic variability among patients, including the dysfunctions of cranial nerves, as well as peripheral motor and sensory defects.

The association between the genetic characteristics and phenotype of patients is not yet clear. Similarly, the influence of those factors on the success of riboflavin is not precisely evaluated. The oral doses of riboflavin needed to achieve a significant improvement in the patient’s condition vary between the cases and lay within the range of 2.0 to 80.0 mg/kg daily [18,22,30]. As of 2024, circa 500 patients worldwide were diagnosed with RTDs [28,29,31,32]. In order to present insight into the differences between the reported cases, a literature search focused on RTD2 reports published in English in the last 15 years was conducted, and the results are presented below in Table 1 [24,33,34,35,36,37,38,39,40,41,42,43,44,45].

In patients with complex neurological symptoms, genetic testing, like the wide gene panel or even whole exome or genome sequencing, is very important to find the core of the disease. These tests give us a wide screen of differential diagnoses including rare neurologic, metabolic or mitochondrial conditions.

Genes such as *SLC52A2* and *SLC53A3* encode the transmembrane proteins (hRFVT2 and hRFVT3, respectively) that mediate the cellular uptake of riboflavin in the intestine and reabsorption in the kidney. Riboflavin, then, is converted to the coenzymes flavin mononucleotide (FMN) and flavin adenine dinucleotide (FAD), which play a crucial role in the transport of electrons in oxidation–reduction cycles. The pathogenic variants in *SLC52A2* and *SLC52A3* reduce riboflavin uptake and riboflavin transporter protein expression, which are the main molecular pathomechanisms of RTDs.

The group of RTDs have similar symptoms to mitochondrial diseases, muscular atrophies (spinal muscular atrophies with or without respiratory distress) and metabolic disorders (MADD), and genetic testing is very helpful to establish the final diagnosis and manage the correct treatment. Here, we have patients with rapid neurologic regression from 3 years of age, who needed a quick diagnosis and treatment. We did not have the possibilities or time to proceed with expression studies in our patients, but the clinical features in the above two patients and the additional family segregation of variants in the *SLC52A2* gene show that one VUS variant in the *SLC52A2* gene, which was not reported before, is probably pathogenic, and diagnosis has been established. The treatment has started with a good outcome at the beginning. Our data indicate that the variant c.477C>G (p.Cys159Trp) is probably pathogenic, and this information could be helpful in future diagnostics for other patients with similar symptoms. It is natural that this variant needs further studies.

The phenotype of the children described in the current report was consistent with the presentations of previous RTD2 cases, though minor differences were apparent. The majority of patients in the reviewed literature were female, which corresponds with the gender of the patients presented in our study. Similarly, both girls are afflicted by a pathogenic mutation in the *SLC52A2* gene, which appears to be more frequently found than other mutations. The mean age at onset was, however, lower than in most reported cases.

The motor development of our patients was delayed, which is not usual for this group (15/44, 34%). Patient 2 also experienced developmental problems in terms of speech, which is even more atypical for RTD2 patients (6/44, 13%). In terms of symptomatology, the deficits present in the patients of the current study were commonly found in the general population of RTD2 patients, mostly resembling the effect of progressive neuronal damage. Ataxia, ataxic gait, dysarthria and nystagmus are present in most patients. Patient 1. and Patient 2. both presented with gait abnormalities. The sensory system of Patient 1. was also affected in terms of hearing and visual impairment, which was in accordance with previous cases. The disorders mentioned above stem from the damage of neuronal cells in the spinal cord and pontobulbar region of brain. Other neurological deficits that reflect the cranial or peripheral nerve function impairment are also frequent, respectively, in terms of bulbar palsy symptoms (dysphagia), facial paresis, tongue weakness and fasciculations, or peripheral, axial and diaphragmatic hypotonia, the latter of which was also frequently associated with respiratory distress. This complication was a frequent reason for hospitalizations in this group, though, fortunately, neither Patient 1. nor Patient 2. had experienced this hazard.

In both of our patients, riboflavin supplementation led to the stabilization of symptoms and a moderate improvement of their condition, which is consistent with some previous reports [46]. Sadly, treatment with riboflavin does not always effectively inhibit the progression of the disease and determines the improvement of the auto-repair process [47]. In the course of RTD, despite taking riboflavin, there are cases of rapid and persistent deterioration, and even sudden deaths, also registered among patients who seem to be stabilized and who are making clear, steady progress, with a so-called good prognosis. This is one of the main reasons for further developing the research (which is now conducted in parallel at several universities and is, fortunately, at a very advanced stage) to develop gene therapy for RTD.

## 4. Conclusions

In summary, despite the low prevalence of RTD2, this disorder should be taken into consideration in the cases of children who are born after an uneventful pregnancy and develop normally, but suddenly begin to experience symptoms of motor delay, as well as other neurological symptoms associated with neuropathies of the cranial and peripheral nerves. The disorder does not necessarily need to manifest itself in laboratory examinations as lowered B_2_ vitamin serum concentrations, which underlines the importance of proactive awareness and the insightful use of genetic assessment. Though children with RTD2 present with a severe, neurological clinical image, vitamin B_2_ supplementation might lead to some improvement, even with minimal applied doses.

## Figures and Tables

**Table 1:** A summary of previous reports of pediatric patients with RTD2.

Authors	Current Study	Total	Cheng et al.	Tranel et al.	Kentab et al.	Sabeghi et al.	Piecuch et al.	Naami et al.	Liu et al.	Carey et al.	Yılmaz et al.	Gayathari et al.	Kranthi et al.	Pillai et al.	Carreau et al.	Fan et al.	Anderson et al.	Woodcock et al.	Nimmo et al.	Thulasi et al.	Shashi et al.	Petrovski et al.	Menezes et al.	Srour et al.	Spagnoli et al.	Haack et al.	Koy et al.	Anand et al.
Year	2024	2012–2024	2024	2024	2024	2024	2023	2022	2021	2021	2021	2021	2020	2020	2020	2018	2019	2018	2018	2017	2015	2015	2016	2014	2014	2012	2012	2012
No. of patients (F—Female; M—Male)	2F	28F 16M	1M	1F	1M	2F	1F	1F	1M	1F	1M	3F 1M	1M	1M	1F	1F	3F 1M	1M 1F	2M	1F	1F	1F	3F 3M	3F 2M	1F	1F	1F	1F
Mean age [years]	2.33	5.73	0.50	16.00	1.50	7.00	4.00	1.08	4.00	18.00	11.00	6.00	10.00	2.00	18.00	2.50	2.38	1.84	0.40	2.50	1.67	1.33	14.50	2.25	0.50	3.00	2.50	1.83
Gene	*SLC52A2*	29x *SLC52A2*; 18x *SLC52A3*	*SLC52A2*	*SLC52A3*	*SLC52A3*	1x *SLC52A3* and *SLC52A2* 1x *SLC52A2*	*SLC52A2*	*SLC52A2*	*SLC52A2*	*SLC52A3*	*SLC52A2*	*SLC52A3*	*SLC52A2*	*SLC52A2*	*SLC52A3*	*SLC52A2*	3x *SLC52A2*; 1x *SLC52A3*	*SLC52A2*; *SLC52A3*	*SLC52A2*; *SLC52A3*	*SLC52A3*	*SLC52A2*	*SLC52A2*	*SLC52A2*	*SLC52A2*	*SLC52A3*	*SLC52A2*	*SLC52A3*	*SLC52A3*
Symptoms																												
Motor delay	++	15	+	-	+	-	-	+	+	-	-	-	-	+	-	-	+++	++	++	-	-	-	-	-	+	+	-	+
Speech delay	+	6	-	-	+	-	+	-	-	-	-	-	-	+	-	-	+	-	+	+	-	-	-	-	-	-	-	-
Ataxia	++	25	+	-	-	+	+	-	-	-	-	+++	-	+	-	+	++	-	+	+	+	-	++++++	+++++	-	+	-	-
Dysphagia	++	26	-	+	+	+	+	-	-	-	+	++++	-	-	-	+	+++	+	-	+	+	-	++++++	++	+	-	-	+
Dysarthria	-	18	-	+	-	+	-	-	-	-	-	++	-	+	-	+	++	-	-	+	-	-	++++++	++	-	-	-	+
Dysphonia	-	2	-	-	-	-	-	-	-	-	-	++	-	-	-	-	-	-	-	-	-	-	-	-	-	-	-	-
Nystagmus	+	9	+	-	-	-	+	+	-	-	-	-	-	-	-	-	++	+	-	-	+	+	-	-	-	+	-	-
Hypotonia	++	34	+	+	+	++	+	-	+	-	+	+++	+	-	+	-	++	++	+	+	+	+	++++++	++++	+	-	+	+
Gait abnormalities	++	12	-	-	+	+	+	-	-	+	-	-	-	-	-	-	-	-	-	+	-	+	-	+++++	-	+	-	-
Polyneuropathy	-	3	-	+	-	+	-	-	-	-	-	-	-	-	-	+	-	-	-	-	-	-	-	-	-	-	-	-
Ptosis	-	9	-	+	-	-	-	-	-	-	-	+++	-	-	-	-	+	-	-	+	-	-	-	-	+	-	+	+
Facial paresis	-	12	-	+	+	-	-	-	-	+	-	++++	-	-	-	-	++	-	-	+	-	-	-	-	-	+	+	-
Optic atrophy	-	15	-	+	-	-	-	+	-	-	-	-	+	-	-	+	-	+	-	-	+	-	++++++	++	-	+	-	-
Visual impairment	+	10	-	-	-	-	+	+	-	-	-	-	+	-	-	-	-	-	-	-	-	-	++++++	-	-	+	-	-
Hearing impairment	+	33	-	+	+	++	+	-	-	+	+	+++	+	-	-	+	++++	++	+	+	+	-	++++++	++++	-	+	+	-
Tongue fasciculations	-	10	-	+	-	+	-	-	-	+	-	+++	+	-	-	-	-	-	-	-	-	-	-	++	+	-	-	-
Tongue weakness	-	7	-	+	-	-	-	-	-	-	-	-	-	-	-	-	-	-	-	-	-	-	++++++	-	-	-	-	-
Respiratory distress	-	16	-	-	+	+	-	-	-	+	+	+	-	-	+	-	++	+	+	+	-	-	++	+	+	-	-	+
Riboflavin treatment (dose; duration)	10 mg/kg daily; continued	-	0.8 mg/kg daily increased to 6.25 mg/kg; 1 week	40 mg/kg daily; 7 months	20–80 mg/kg/d; 22 months	600–900 mg/day; 3 months	No information	80 mg/kg daily; 6 weeks	1.7 mg/kg daily; 2 weeks	1.0–1.5 g/daily; 3–6 months	75 mg/kd daily; 3 weeks	100–400 mg/twice a day	25 mg/kd daily; 6 months	70 mg/kg daily; 11 months	15 mg/kg daily; 1 year	4.5–13 mg/kg daily; 6 years	10–70 mk/kg daily; 1–5 years	50 mg/kg daily; 3 months	70–80 mg/kg daily; 2–12 months	25–60 mg/kg daily; 3.5 years	10–70 mg/kg daily; 2 months	10–70 mg/kg daily; 2 months	1000 mg daily; 12 months	10–15 mg/kg daily; 3 months	150 mg daily; 4 years	10 mg/kg; 4 weeks	10 mg/kg; 1 week	25 mg/kg; 7 months
Therapy success	Yes	-	Partial	Yes	Yes	Partial	No information	Yes	Yes	Partial	Yes	Yes	No	Partial	Partial	Partial	Partial to significant	Partial	Partial	Yes	Yes	Yes	Partial	Partial	No	Yes	No	Yes

## Data Availability

The data presented in this study are available on request from the corresponding author.

## Supplementary Materials

The following supporting information can be downloaded at: https://www.mdpi.com/article/10.3390/metabo15110688/s1, Figure S1: Sanger sequencing results of *SLC52A2* gene of patient 2.

## Abbreviations

RTD	Riboflavin transporter deficiency
FMN	Flavin mononucleotide
FAD	Flavin adenine dinucleotide
RTDs	Riboflavin transporter deficiencies
BMI	Body mass index
MRI	Magnetic resonance imaging
USG	ultrasonography
