# Supplementary figures and images for: Riboflavin Transporter Deficiency as a Cause of Progressive Encephalopathy
Source: Metabolites. 2025 Oct 24;15(11):688. doi: 10.3390/metabo15110688 (PMC12654560; doi:10.3390/metabo15110688)

# Supplement Figure S1

Sanger sequencing results of *SLC52A2* gene of patient 2

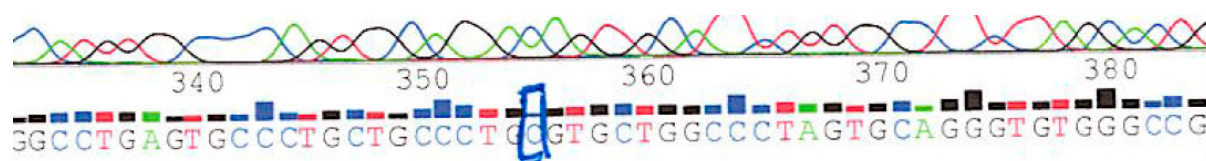

c.477C>G (p.Cys159Trp)

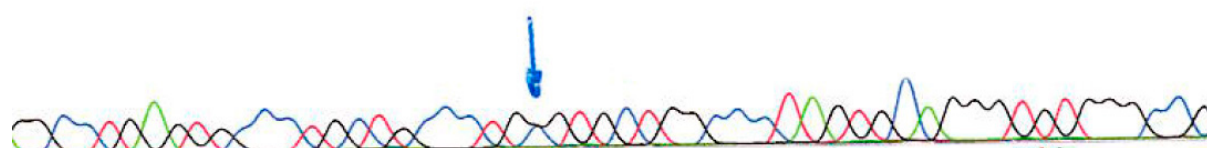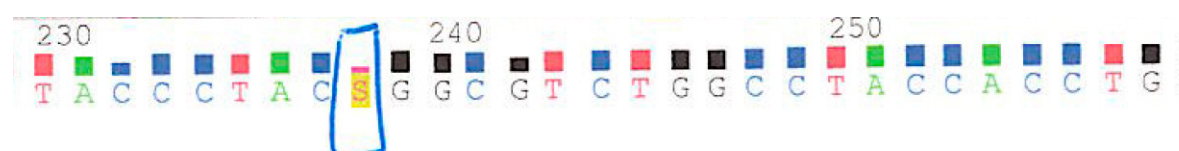

c.916G>C (p.Gly306Arg)

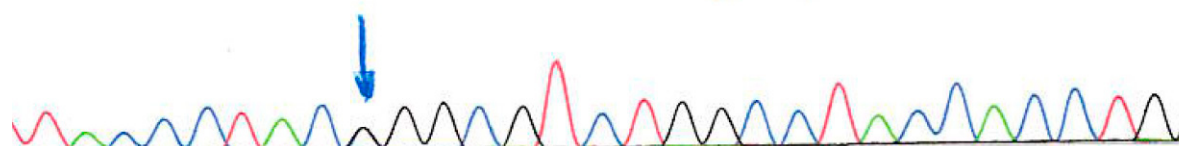

Supplement: Supplementary file 1 [file metabolites-15-00688-s001.zip › metabolites-3850387-supplementary.pdf]
